# Analyzing Effects of Naturally Occurring Missense Mutations

**DOI:** 10.1155/2012/805827

**Published:** 2012-04-22

**Authors:** Zhe Zhang, Maria A. Miteva, Lin Wang, Emil Alexov

**Affiliations:** ^1^Computational Biophysics and Bioinformatics, Department of Physics and Astronomy, Clemson University, SC 29634, USA; ^2^Université Paris Diderot, Sorbonne Paris Cité, Molécules Thérapeutiques In Silico, Inserm UMR-S 973, 35 rue Helene Brion, 75013 Paris, France

## Abstract

Single-point mutation in genome, for example, single-nucleotide polymorphism (SNP) or rare genetic mutation, is the change of a single nucleotide for another in the genome sequence. Some of them will produce an amino acid substitution in the corresponding protein sequence (missense mutations); others will not. This paper focuses on genetic mutations resulting in a change in the amino acid sequence of the corresponding protein and how to assess their effects on protein wild-type characteristics. The existing methods and approaches for predicting the effects of mutation on protein stability, structure, and dynamics are outlined and discussed with respect to their underlying principles. Available resources, either as stand-alone applications or webservers, are pointed out as well. It is emphasized that understanding the molecular mechanisms behind these effects due to these missense mutations is of critical importance for detecting disease-causing mutations. The paper provides several examples of the application of 3D structure-based methods to model the effects of protein stability and protein-protein interactions caused by missense mutations as well.

## 1. Introduction

Human DNA is not identical among individuals, and this causes natural differences among races and ethnic populations, and also among healthy individuals and individuals susceptible to disease. On the DNA sequence level, the differences could be large or small, the smallest being a difference in a single-nucleotide. If such a difference occurs in some fraction of the population, but not in a single case, the difference is termed single nucleotide polymorphism (SNP) [1, 2]. Some of the SNPs occur at the noncoding, while other SNPs happen in the coding regions [3]. The SNPs occurring at the noncoding region does not affect the gene product, that is, the protein sequence is not changed, such types of SNPs are termed silent mutations [4]. However, silent mutation could also be found in the coding region because each amino acid is coded by more than one codon. Thus, even the mutation changes the codon, though it is still possible that the protein sequence is not affected. However, a silent mutation still could affect the function of the cell by altering the gene's expression and regulation.

On the other side of the spectrum are nonsynonymous SNPs (nsSNPs), which cause changes in protein sequence. The most dramatic change is induced by the nonsense mutations, which result in a premature stop codon and produce truncated, usually nonfunctional proteins [4]. Missense mutation, on the other hand, is a change of a single amino acid into another. Such a mutation could be polymorphism if it is observed in significant fraction of the population, or it could be a rare missense mutation if found in an individual or small group of people, as, for example, in a family. In both cases, on protein level, these mutations are termed single-point mutations and they are the primary focus of this paper.

Missense mutations and, in general, nsSNPs were extensively investigated in the past to reveal their plausible effects on protein stability [5–10], protein-protein interactions [11], the characteristics of the active site [5, 6], and many others [12–20]. In parallel, significant efforts were invested to catalog naturally occurring genetic differences, those found in general population and presumably harmless as the SNP database [21–23] and those known to be disease associated as the Online Mendelian Inheritance in Man (OMIM) [24–26]. The OMIM includes the full-text description of disease phenotypes and genes, mapping, molecular genetics, PubMed references, and many other features [26, 27]. OMIM is currently provided by the U.S. National Center for Biotechnology Information (NCBI) [28] and edited by Dr. Victor A. McKusick at John Hopkins University. By 2011, more than 21,000 entries including data and over 13,100 established gene loci and phenotypic descriptions are contained in OMIM Entrez database [28]. SNP database is used to organize and systematize the huge amount of information of gene sequencing. So far, several SNP databases have been developed such as the dbSNP database [21, 29, 30], the Human Genome Variation Database (HGVbase) [22], the Human Gene Mutation Database (HGMD) [31], and the TopoSNP database [23, 32–35].

The existence of such databases combined with available biochemical data of the effects of single-point mutations on protein stability and interactions prompted the development of *in silico* methods to predict the effects of mutations on the wild-type characteristics of the corresponding proteins or assemblages. Currently, the approaches can be classified into several categories: first principle methods, which calculate the folding or binding free energy change based on detailed atomic models [36–46]; methods based on statistical potentials [47–55] and utilizing known protein structures in the Protein Data Bank [56]; methods using empirical potential combining both physical force fields and free parameters fitted with experimental data [57–65]; machine learning approaches, which are trained against known experimental databases, and then used to predict the effect of the newly found mutations [66–72].

## 2. Overview of Plausible Effects Induced by Genetic Differences

Genetic differences can potentially affect the function of the cell in a variety of ways, which can be broadly classified into several categories outlined below.

### 2.1. Active Sites, Reaction Kinetics, and the Reaction Parameters

If a mutation occurs in an active site, then it should be considered lethal since such substitution will affect critical components of the biological reaction, which, in turn, will alter the normal protein function [73, 74]. At the same time, the biochemical reaction is very sensitive to the precise geometry of the active sites for both of the reactants and products; therefore, any conformational change altering the active sites will also affect the biochemical reaction; however, conservative mutations are not expected to perturb protein function by much. Thus, even if the mutation does not occur at the active site, but quite close to it, the characteristics of the catalytic groups will be perturbed [5, 6, 75]. In such a case, the mutation may not completely abolish the biochemical reaction but can change the kinetics of the reaction [76]. Moreover, the biochemical reaction strongly relies on a particular (optimum) cellular environment such as pH, salt concentration, and temperature. Thus in the living cells, the proteins' behavior is controlled by these cellular environments [77, 78]. Changing the reaction rate, the pH, or salt and temperature dependencies away from the native parameters can lead to a malfunctioning protein. The isoelectric point (pI) is a very important parameter that refers to the pH at which the net charge of the protein is zero. Recently, it was demonstrated that five missense mutations involving charged groups in the sodium iodide symporter (NIS) gene, which generates a protein called iodide transporter and is associated with iodide transport defect, can cause an obvious pI shift and influence the electrostatic interactions in the trans-membrane domains of the NIS protein. Even more, these substitutions will probably, in turn, affect the protein stability, protein trafficking, and iodide transport activity [79].

### 2.2. Kinetics of Protein Folding, Protein Stability, Flexibility, and Aggregation

Protein folding is the process of converting the linear unfolded polypeptide into the native 3D structure driven by the gradient of potential energy [80, 81]. The importance of kinetics of protein folding is manifested by the fact that protein miss-folding is involved in many diseases [12]. An amino acid substitution at a critical folding position can prevent the forming of the folding nucleus, which makes the remainder of the structure rapidly condense [12]. Protein stability is also a key characteristic of a functional protein [5, 6, 71, 82–86], and as such, a mutation on a native protein amino acid can considerably affect its stability [76, 77, 87] through perturbing conformational constraints (e.g., substituting a small side chain residue to a large one and vice versa, resulting in backbone strain or overpacking) or physicochemical effects (substitutions between hydrophilic residues and hydrophobic residues, burial of charged residues, the disruption of hydrogen bonds, loss of hydrogen bonds, of S–S bonds) [88]. It was shown that 80% of missense mutations associated with disease are amino acid substitutions that affect the stability of proteins by several kcal/mol [84]. In addition, the missense mutation can also alter the protein flexibility [5, 89, 90]. When a protein is carrying its function, frequently the reaction requires a small or large conformational change to occur that is specific for the particular biochemical reaction. Thus, if a mutation makes the protein more rigid or flexible compared to the native structure, then it will affect the protein's function [91, 92]. Additionally, conformational flexibility is the main mechanism affecting protein aggregation propensity [93], thus the influence on protein flexibility could cause protein aggregation and formation of fibrils [94].

### 2.3. Interactions between Protein-Protein, Protein-DNA, Protein-RNA, and Protein-Membrane

If a missense mutation occurs at hot-spots of the binding interface that are crucial in contributing to the interaction [95, 96], then the binding affinity would be dramatically affected due to geometrical constrains and/or energetic effects [7, 97]. For instance, when substituting a small side chain for a bulky side chain in a narrow binding pocket, the entrance of the partner group will be blocked and the binding process will be completely or partially prevented [6, 98–101]. Similarly, a mutation at the protein-DNA interface can affect DNA regulation [13–15]. A mutation occurring at the protein-membrane interface can affect the signal processes across the membrane, protein association with the membrane, and function of various channels and pumps [16, 17].

### 2.4. Subcellular Localization and Protein Expression

Subcellular localization is a very important factor, which provides a specific environment for protein function, protein interactions, protein activity in signaling pathways, and many other features. Transporting a protein to the correct compartment allows it to form the necessary wild-type interactions with its biological partners and take part in the corresponding biological networks like signaling and metabolic pathways. Otherwise, mislocalizing the protein in a wrong subcellular compartment will have harmful effects on the other proteins which function there [20]. Typically, a mutation affecting the subcellular localization is a mutation that occurs at a signaling region. For example, missense mutations in Otopetrin 1 affects the subcellular location and causes nonsyndromic otoconia agenesis and a subsequent balance defect in mice [102]. Fanconi anemia is a genetic disease associated with the missense mutations in FANCA protein. These missense mutations affect the subcellular localization of the FANCA protein and make it unable to relocate to the nucleus and activate the FA/BRCA pathway [103].

Protein expression is a subcomponent of gene expression and commonly used to denote the measurement of the protein concentration in a particular cell or tissue. Missense mutations can affect DNA-transcription factors resulting in altering the expression of the corresponding protein. Altering the wild-type protein expression in the compartment where it is designed to function will disrupt the normal cell cycle and in turn may cause diseases [20]. Recently, functional analysis of pancreatitis-associated missense mutations was performed in the pancreatic secretory trypsin inhibitor (SPINK1) gene, which encodes pancreatic secretory trypsin inhibitor (PSTI). It was shown that one of the disease-causing missense mutations R65Q reduced protein expression by almost 60%, and four other pathogenic missense mutations G48E, D50E, Y54H, and R67C caused complete or almost complete loss of PSTI expression [104]. By excluding the possibility that reduced transcription or unstable mRNA can lead to reduced protein expression, it was surmised that these disease-causing missense mutations probably cause intracellular retention of their respective mutant proteins. This is suggestive of a potential unifying pathological mechanism underlying both the signal peptide and mature peptide mutations [104].

In this section, we presented the plausible effects which mutations can cause. In fact, mutations often affect the normal protein function by the combined molecular effects listed above [5, 6, 8]. For Instance, in the studies of genotype-phenotype correlations of TGFBI (transforming growth factor, beta-induced) mutations, it was shown that a missense mutation V613G strongly destabilizes the wild-type protein keratoepithelin by 3.1 kcal/mol; additionally, the same mutation might also result in an improper folding due to the backbone structure of the substituted gly is not restricted by the presence of a side chain, thus can adopt any conformation and lead to a misfolded protein. At the same time, it was shown that V613G also facilitates formation of beta-sheet structure of TGFBI which is known to favor amyloid formation [105]. Similarly, another study performed *in silico* investigation on 18 missense mutations in electron transfer flavoprotein (ETF) associated with multiple acyl-coa dehydrogenase deficiency (MADD), and it was found that these 18 missense mutations can be classified into two groups by their molecular effects: altering protein folding and assembling, affecting the catalytic activity of functional sites, and disrupting interactions with their biological partner, that is, dehydrogenases in this case [106].

## 3. Methods and Approaches to Predict the Effects of Mutations

Current efforts in this field are aimed at predicting the deleterious mutations since such predictions can be used for diagnostics and drug design. The features used to make such predictions can be classified into three categories: (a) amino acids properties, such as size, side chain polarity, side chain flexibility, and its ability to form a hydrogen bond and other geometrical considerations; (b) 3D protein structural properties such as protein stability, affinity of receptor-ligand complex, and structural flexibility; (c) evolutionary properties like sequence conservation and phylogenetic trees. It is almost impossible to review these approaches one by one since most of the current methodology is using a combination of these features [27].  Table 1 shows several examples for application of molecular modeling methods, free of charge for academia, to study the molecular mechanisms of missense mutations affecting wild-type properties of proteins. Comparison of their performance is provided in references [65, 107]. In the following paragraphs, we explain in detail some of the available resources.

It is essential to identify the most informative features among the features mentioned above for making successful predictions. Such a necessity inspired several works among which a recent study evaluating 32 features using their mutual information together with the functional effects of the amino acid substitutions, as measured by *in vivo* assays. Sequentially, a greedy algorithm was performed to identify a subset of highly informative features [108]. Finally, it was concluded that two features describing the solvent accessibility of  “wild-type” and “mutant” amino-acid residues and another feature of evolutionary properties based on superfamily-level multiple alignments produce the best accuracy [109]. Another investigation developed a formalism and a computational method based on a structural model and phylogenetic information to indicate the effects of amino acid substitution on protein functions. With such a protocol, approximately 26%–32% of naturally occurring missense mutations were predicted to affect the protein functions [110].

The amino acid properties are often considered an important characteristic, which could play a crucial role in protein folding, stability, interaction of protein-protein complexes, and protein function, although sometimes they may be misleading [11]. The side chain properties such as volume, polarity, acidity, basicity, conformational flexibility and the ability to form a hydrogen-bond and salt bridge, are distinguishable. Therefore, the compatibility of a substitution at the dominant allele could be used to make the prediction as it was done in a recent study [111], which combined amino acid properties and structural information to identify deleterious mutations by analyzing the effects on protein stability.

An alternative approach to assess the effect of mutation on protein stability is to evaluate the change of folding free energy ΔG(folding). The difference between ΔG(folding) of the wild-type protein and the mutant, typically described as ΔΔG(folding), is a measure of the effect of mutation on protein stability [5, 6, 64, 65, 107, 112]. If the change in ΔΔG(folding) is negative, then the prediction is that the mutation will destabilize the protein. In contrast, if the calculated change is positive, the mutation is expected to stabilize the protein. The same considerations are valid in the case of predicting the effect on receptor-ligand binding [113]. Numerous investigations were reported in the past to reveal the change in the stability of the native structure [71, 82–86], the macromolecular interactions [11], or altering the wild-type (WT) hydrogen-bond network, in terms of affecting the stability [5, 114, 115]. Currently, several distinctive approaches to predict the protein stability and affinity changes due to mutations have been developed and they can be classified into four categories: (a) first principle methods that using the detailed atomic models to calculate the folding/binding free energy changes caused by mutations [36–46]—these approaches are scientifically sound, but are quite computationally expensive and may not be the best choice in the cases of large sets of mutations [116]; (b) methods based on the statistical potentials [47, 48] were shown to be successful in predicting the change of protein stability upon the mutations [49–55]; (c) Methods utilizing empirical potential, combining both physical force fields, and free parameters fitted with experimental data [57–62]; (d) machine learning methods, utilizing a training database [66–70].

The 3D structure of proteins can be used not only for energy calculations, as described above, but to map mutations onto it and to use geometrical considerations to predict the effects of mutations [117]. Recently, such an approach, the alpha-shape method from computational geometry, was used to divide all nsSNP sites into three categories: (a) Type P: nsSNPs located in a pocket or a void; (b) Type S: nsSNPs occurred on a convex region; and (c) Type I: nsSNP sites are completely buried inside the protein. It was found that 88% of pathogenic nsSNPs are of type P and rarely of type I [32]. Along the same line, 3D structures were used in combinations with machine learning (SVM) and random forest methods. It was demonstrated that these methods outperformed the SIFT algorithm developed by Ng and Henikoff [118], and was indicated that incorporating structural information is crucial to make an accurate prediction if no sufficient evolutionary information is available [119]. Based on the 3D structures, the solvent-accessibility term is also an important feature, which is often used for investigating the effects of missense mutations. It has been shown that using a solvent-accessibility term, the C*β* density, and a score derived from homologous sequences will make the most accurate prediction [120]. Recent studies took into account several protein structural parameters such as solvent accessibility, location within beta strands, or active sites to predict the effects on nsSNPs. It was found that approximately 70% of the disease-associated mutations are buried and solvent inaccessible [121–124] and that such mutations have strong effects on protein structure, folding, stability, and normal function [121, 123].

Another important feature reviewed here is evolutionary properties. Among homologous proteins, the highly conserved residues are generally considered to be critical for protein stability, interaction, and function. One of the evolutionary approaches, which assumes that residues located at a highly conserved position are most likely crucial, is to extract conservation scores from a multiple sequence alignment of homologous proteins. Another widely used computational technique is named the “evolutionary trace” method [125–127]. It uses phylogenetic information based on homologous sequences to rank residues according to evolutionary importance based on their conserved residues in the protein family. After that, such evolutionary conserved residues are mapped on the representative structure. In addition, a group of conserved residues could occur at the interface of a protein-protein complex. Based on the extraction of functionally important residues, an approach was developed utilizing the evolutionary trace method to identify active sites and functional interfaces of proteins based on their available structures. The method was tested on SH2 and SH3 modular signaling domains and the DNA binding domain of the hormone receptors. It was demonstrated this method can delineate the functional epitope and identify the essential residues for binding specificity [128].

In order to train machine learning algorithms properly and to have a benchmarking case, appropriate databases are required. A particular example is the Catalytic Site Atlas database [129], which collects 177 original hand-annotated entries and 2608 homologous entries and covers about 30% of all enzyme in the Protein Data Bank [56]. At the same time, the computational methods of predicting the functional residues were also well-developed [125, 130, 131]. A selection of structure and sequence-based features was used to indicate an amino acid polymorphism effect on protein function, and it was found that ~26%–32% of the naturally occurring nsSNPs will affect the protein's function [110].

## 4. Webservers for Analyzing the Effects of Mutations

In past years, several methods were implemented into webservers to predict the effects on protein stability due to mutations. The Eris webserver is based on Medusa force field [132], and it was benchmarked on 595 mutants with available experimental data resulting in RMSD 2.4 kcal/mol between the predicted ΔΔG_cal_ (folding) and corresponding experimental values (ΔΔG_exp_ (folding)) [112, 132, 133]. The FoldX is perhaps the most popular web server [63] for predicting the folding free energy changes due to the mutations, and it is based on the empirical potentials [64]. The I-Mutant 2.0/3.0 is Support Vector Machine-based (SVM: a machine learning method) webserver utilizing the 3D structural or sequential information to predict protein stability change upon single-point mutations [71, 72, 134]. Other webservers include the Site Directed Mutator (SDM) [54] and the Mupro method [135].

In parallel, there are webservers predicting the effects of mutations on protein-protein interaction. The COILCHECK is an interactive webserver, which measures the strength of interactions between two helices involved in coiled coil structures utilizing nonbonded and electrostatic interactions and the presence of hydrogen bonds and salt bridges. It can be used to assess the strength of coiled coil regions, to recognize weak and strong regions, to rationalize the phenotypic behavior of single mutations and to design mutation experiments [136]. Recently, DrugScore^PPI^ was reported, which is a fast and accurate computational approach to predict impacts on binding affinity by the change of the binding free energy upon alanine mutations at protein-protein interfaces. The primary motivation of developing this webserver is to identify hotspot residues at protein-protein interfaces, which will guide both biological experiments and the development of protein-protein interaction modulators [137].

There are many webservers Which are designed to predict if the mutation is pathogenic or not without providing information about the magnitude of expected energy changes. The SNPs3D [138] is a primary resource and database, which provides various disease/gene relationships at the molecular level. This server has three modules: (a) identifying the gene candidates involved in a specific disease; (b) relationships between the sets of candidate genes; and (c) analyzing the possible effects of nsSNPs on normal protein function. It is very convenient for the users to quickly obtain the available information and so develop models of gene-pathway-disease interaction. Another online predictor of molecular and structural effects of protein-coding variants was recently developed, the SNPeffect 4.0 [139]. It uses sequence- and structure-based bioinformatics tools such as aggregation prediction (TANGO) [140], amyloid prediction (WALTZ) [141], chaperone-binding prediction (LIMBO) [142], and protein stability analysis (FoldX) [63] to predict the effect of SNPs. In addition, it also contains the information of effects on catalytic sites, posttranslational modifications, and all known human protein variants from Uniprot. At the same time, SNPeffect allows users to submit custom protein variants for analyzing the SNP effects and plot correlations between phenotypic features for a user-selected set of variants [139]. The dbSNP database in NCBI lists over 9 million SNPs in the human genome but includes very limited annotation information. To fill this gap, the LS-SNP was developed to annotate the nsSNPs [83]. It can map nsSNPs onto protein sequences, functional pathways, and comparative protein structure models and predicts the positions where nsSNPs cause the effects. The results can be used to find out the functional SNP candidates within a gene, haplotype, or pathway, and also in understanding the molecular mechanisms responsible for functional effects of nsSNPs [83]. At the same time, a protocol based on Sorting Intolerant From Tolerant (SIFT) [118] was reported to predict if a missense mutation will affect the protein function. To assess the effects of a missense mutation, SIFT utilizes evolutionary properties of the protein and considers the substitutions at the conserved positions which may affect protein function. Thus, SIFT makes a prediction on effects of all possible substitutions at each position in the protein sequence by using sequence homology [143]. The Polyphen (Polymorphism Phenotype) is a tool that predicts possible impact of an amino acid substitution on the structure and function of a human protein using straightforward physical and comparative considerations. It combines a variety of features such as sequences, evolutionary properties, and structural information to predict if an nsSNP will affect the protein function and performs optimally if the structural information is available. More than 11000 nsSNPs are annotated by this webserver [86]. A new version of Polyphen, namely, Polyphen-2, was recently released [144]. Its features include high quality multiple sequence alignment pipeline and probabilistic classifier based on a machine-learning method, and it is optimized for high-throughput analysis of the next-generation sequencing data [144]. After the development of SIFT and Polyphen, the Parepro (Predicating the amino acid replacement probability) was created, based on two independent databases HumVar and NewHumVar. The predictions are if an nsSNP will be either deleterious or will have no effect on protein function. Compared to SIFT and Polyphen, Parepro achieved a higher Matthews correlation coefficient (MCC) and overall accuracy (Q2) when predications were made using a 20-fold cross validation test on the HumVar dataset [145]. StSNP is a webserver referencing the data from dbSNP in NCBI, the gene and protein database from Entrez, the protein structures from the PDB, and pathway information from KEGG and makes an effort to provide combined, integrated reports about nsSNPs. Researchers can use the metabolic pathways in StSNP to examine the likely relationship between the disease-related pathways and particular nsSNPs, and link the disease with the current available molecular structure data [146]. AUTO-MUTE is a knowledge-based computational mutagenesis used to predict the disease potential of human nsSNPs. In this study, 1790 neutral and disease-associated human nsSNPs on 243 diverse human protein structures were used. With a trained model, this method achieves 76% cross-validation accuracy [147].

## 5. Application of Structure-Based Methods to Predict the Effects of Mutations on Protein Stability and Protein-Protein Interactions

In this section, we outline several examples of utilizing structural information to predict the effects of mutations on wild-type characteristics of proteins and protein complexes.

### 5.1. Application of Molecular Dynamics (MD) Simulation for Predicting the Effects of Mutations

**Figure 1:**
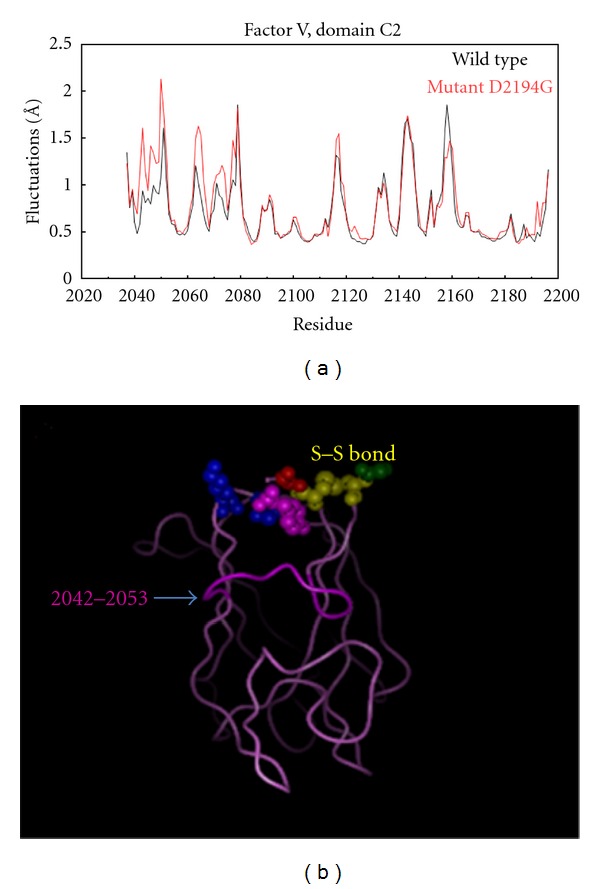
(a) The structural and flexibility differences between the simulated WT and mutant structures. The black line represents the RMSF of the WT structure and the red line represents the mutant protein. (b) 3D structure of the C2 domain of the WT FV. The S–S bond is marked in yellow and the loop 2042–2053 is indicated by the arrow.

Coagulation factor V (FV) is the precursor of an essential procoagulant cofactor that accelerates FXa-catalyzed prothrombin activation in the coagulation system. It is a large glycoprotein containing several domains, A1-A2-B-A3-C1-C2 [8]. A missense mutation D2194G in its C2 domain was shown to cause low expression level and to have plausible effect on stability of the corresponding protein. To investigate the molecular mechanism of D2194G affecting the wild type of the corresponding protein, MD simulations were carried out on both of the WT and mutant structure to reveal the flexibility change upon this mutation [8]. The program CHARMm [148] was used, and the total simulation time was 900 ps. The root mean square fluctuations (RMSFs) for the *α*-carbon atoms of the C2 domain per residue were calculated for series of snapshots. The comparison for the WT and mutant structures is shown in Figure 1(a). It was concluded that the regions 2075–2085 and 2140–2150 in both WT structure and mutant structures are flexible. The loop 2042–2053 (Figure 1(b)) which is close to the mutation site, is more flexible in the mutant structure. At the same time, loop 2060–2067 became more flexible in the mutant as well, and this effect was attributed to the increased mobility of the loop 2042–2053. The substitution of Asp for Gly will lead to a big cavity and the nonflexible C-terminus (Tyr2196) inserts itself into the domain and attempts to fill out this cavity and to compensate for the missing negative charge of the mutant. These events could be the reason for the enhancing flexibility of the loop 2042–2053.

### 5.2. Application of Energy Calculation for Predicting the Effects of Mutations in Human Spermine Synthase

In this section, we describe the molecular mechanism of three missense mutations in human spermine synthase (SMS) causing Snyder-Robin Syndrome (SRS) [149–151] to demonstrate application of structure-based methods and energy calculation to predict the effects on protein stability and protein-protein interaction [5].

**Figure 2:**
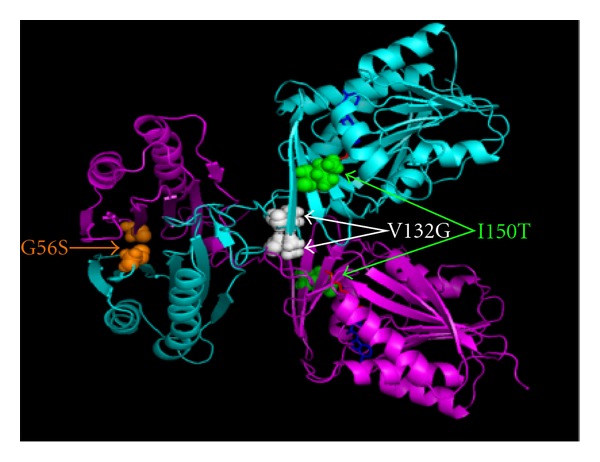
3D structure of human SMS with three missense mutation sites. Two subunits were represented by ribbon in cyan and magenta. Three mutation sites were shown with sphere representation: G56S in orange, V132G in white and I150T in green. The substrates of SPD and MTA were shown in red sticks and blue sticks, respectively.

SMS (OMIM: 300105) is an enzyme converting spermidine (SPD) into spermine (SPM) both of which are two polyamines controlling normal mammalian cell growth and development [152–155]. The importance of SMS for the normal function is illustrated by the fact that three clinical missense mutations, c.267G > A (p.G56S) [150], c.496T > G (p.V132G) [151], and I150T [5], on SMS will cause an X-Linked mental retardation disorder named SRS (OMIM: 309583). At the same time, the 3D structures of human SMS with either the substrates SPD or product SPM have been experimentally determined [156]. The 3D structure of SMS with the substrates SPD and product MTA (PDB ID: 3C6K) is shown in Figure 2. SMS contains two subunits forming a dimer, and each subunit includes two terminal domains: the N-terminal domain which plays a key role in dimerization and the C-terminal domain which includes the active site. The importance of dimerization for SMS function was also demonstrated by series of deletion experiments *in vitro* [156]. Additionally, two missense mutations G56S and V132G are located at the dimer interface, while the other missense mutation I150T occurred at the C-terminal domain and quite close to the active sites.

**Figure 3:**
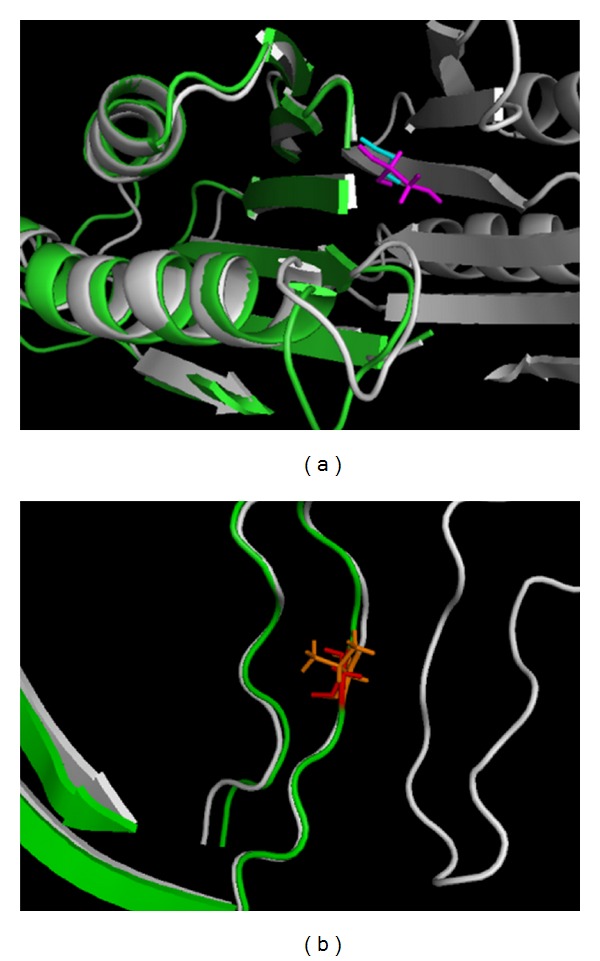
Effects on dimerization. (a) G56S: we superimposed WT structure (presented with two chains in white) and mutant structure (presented with Only one chain in green). Cyan stick represented Gly in the WT structure and magenta stick represented Ser in the mutant structure; (b) V132G: Only the region around the mutation site was shown in the figure. We superimpose the WT structure (presented with two chains in white) and mutant (presented with only one chain in green). The orange stick represented Val in the WT structure and red stick represented Gly in the mutant structure.

These three mutants were made *in silico* by SCAP, a program in JACKAL package [157], based on the native 3D SMS structure. Then, the TINKER package was used to perform the energy minimization and calculation [158]. It was shown that the missense mutation G56S will strongly decrease the dimer affinity by nearly 14 kcal/mol, but the other two have no impact on it. With the analysis based on the 3D structure, it was concluded the reason that G56S strongly decreases the dimerization is because the side chain of Ser in the mutant is pointing to the dimer interface, and there is no enough room to harbor this side chain (Figure 3(a)). In contrast, while the mutation V132G is located at the dimer interface as well, the side chain of Val in the native structure does not point towards the interface and is close to large cavity (Figure 3(b)); thus this substitution can be accommodated easily without introducing any steric constrains. The third mutation, I150T, is very far away from the dimer interface (Figure 2); thus it is not supposed to affect the dimerization.

**Figure 4:**
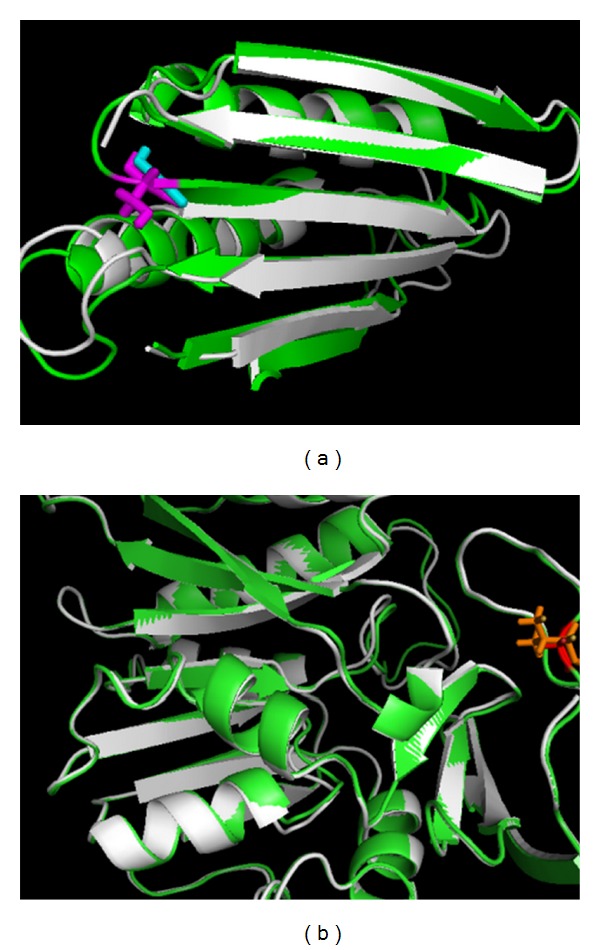
Effects on monomer stability. (a) G56S: N-terminal domain of both WT monomer (white) and mutant monomer (green) are superimposed. Cyan stick represented Gly in the WT structure and magenta represented Ser in the mutant structure; (b) V132G: C-terminal domain of both WT monomer (white) and mutant monomer (green) are superimposed. We use stick and ball representation in orange to represent Val in the WT structure and in red to represent Gly in the mutant structure.

With regards the folding energy calculation, all these missense mutations are predicted to destabilize the protein monomer by 2.8 kcal/mol (G56S), 1.1 kcal/mol (V132G), and 3.5 kcal/mol (I150T), respectively.  Figure 4(a) gives the comparison of the native structure and mutant structure and is zoomed into the mutation site G56S. In the mutant structure, we can see this mutation occurs in a sharp turn, and the substitution with almost any other amino acid will introduce strain.  Figure 4(b) shows the superposition of the native structure and mutant, zoomed in the mutation site V132G. It is clear that the side chain of Val points to the interior, thus the substitution with Gly will leave a big cavity inside the monomer, which in turn will affect the stability. In addition, considering the physicochemical property feature, Val and Gly have different hydrophobicity. The destabilization by I150T is mainly attributed the totally different physicochemical properties between Ile, which is a hydrophobic residue, and Thr, which is a hydrophilic residue.

Thus, combining the 3D structure, physicochemical properties of amino acids, and energy calculations, it was shown that one can successfully predict molecular effects due to these three missense mutations. Such an analysis helps better understand how these missense mutations affect the SMS function and in turn reveal the molecular origin of SRS.

## 6. Conclusion

In this paper, we outlined the current state-of-the-art methods in the field of computational modeling of effects of nsSNPs and rare missense mutations. Available resources are pointed out along with short description of their functionality and accuracy. The basic concepts and major research directions are described and their advantages and disadvantages discussed.

## Figures and Tables

**Table 1:** 

Methods	Short Summary	Examples (references)*	Some tools based on this method
Molecular dynamics	The trajectories of molecules are determined at atomic level by numerically solving the Newton's equation of motion	(i) Thrombosis-related R2-FV haplotype: D2194G, Coagulation Factor V, domain C2 [8] (ii) Parahemophilia, Factor V new brunswick: A221V, Coagulation Factor V, domain A [9] (iii) FPLD, R482W; Lamin A/C [159] (iv) Intellectual Disability: H101Q; CLIC2 protein [10] (v) Snyder-Robin syndrome: G56S, V132G, I150T; spermine synthase; [5]	Eris [112, 132, 133] Tinker [158] GROMACS [160]

Molecular mechanics	Using molecular mechanics force field and optimization to model molecular systems	(i) 21-Hydroxylase-Deficiency: R132C, R149C, M283V, E431K; CYP450; C21 [161] (ii) Cancer: A159V, A161V, N235I, N239Y, T256I, S269I; p53 [162] (iii) Intellectual Disability: H101Q; CLIC2 protein; [10] (iv) Mutability of human spermine synthase: all amino acids substitution at disease associated missense mutation sites G56, V132, and I150; human spermine synthase [6] (v) Studying effects of nsSNPs on protein-protein interactions: nsSNPs in OMIM and non-OMIM; 264 protein-protein complexes with known nsSNPs located at the interface; [11]	FoldX [63, 64]

Monte Carlo simulation	Applying Monte Carlo sampling to predict preferred conformational states	(i) Noonan syndrome: D61Y, Tyrosine phosphatase SHP-2 [163]	IMC [164]

Electrostatic calculation	Calculating electrostatics energy and pKa/ionized states changes/electrostatic stability upon the missense mutations	(i) Snyder-Robinson Syndrome:; G56S, V132G, I150T human spermine synthase [5] (ii) Thrombosis-related R2-FV haplotype: D2194G, Coagulation Factor V, domain C2 [8] (iii) Noonan syndrome: D61Y, Tyrosine phosphatase SHP-2 [163] (iv) Studying effects of nsSNPs on protein-protein interactions: nsSNPs in OMIM and non-OMIM; 264 protein-protein complexes with known nsSNPs located at the interface; [11]	DelPhi [165] MCCE [166–168] pKD [169]

Evolutionary properties	Based on structure and sequence analysis, for example, highly conserved residues in a protein family	(i) Homocystinuria: 204 mutations; cystathionine beta synthase; [170]	SNPs3D [138] PolyPhen [86]

Machine learning	learn the behavior of a system based on training datasets	(i) Snyder-Robinson Syndrome: G56S, V132G, I150T; human spermine synthase; [5] (ii) Gastrointestinal stromal tumors: 19 mutations; KIT receptor [171]	I-Mutant 2.0/3.0 [71, 72, 134]

Graph methods	A branch of discrete mathematics. In protein science, this method is used to analyze the topological details of proteins with known structure	(i) Cancer: Y220C, R273H, R273C, R282W, and G245S; p53 protein; [172] (ii) Predicting the structural effects of nsSNPs: 506 disease-associated nsSNPs; [173]	Bongo [173]

Statistical Potential	Based on the knowledge of statistical mechanics such as inverse Boltzmann law, ΔG = −*kT *log [g_ij_(**r**)]	(i) Human X-linked Agammaglobulinemia (XLA): 16 missense mutations; Bruton's tyrosine kinase (Btk); [174, 175] (ii) Severe form of phenylketonuria: G46S; human phenylalanine hydroxylase (hPAH); [176]	DFIRE [55, 177, 178] PoPMuSiC-2.0 [179, 180] CUPSAT [181–183]

The BellKor collaborative filtering (CF) algorithm	Model relations of the known data points and the parameters of the model are learnt by the training database	(i) Using the known ΔΔG value to predict the ΔΔG value of other missense mutations at the same substitution site; 4803 mutants were used; [184]	Pro-Maya [184]
